# Curriculum Innovation: A Standardized Experiential Simulation Curriculum Equips Residents to Face the Challenges of Chief Year

**DOI:** 10.1212/NE9.0000000000200138

**Published:** 2024-07-12

**Authors:** Elina Zakin, Nada Abou-Fayssal, Aaron S. Lord, Aaron Nelson, Sara K. Rostanski, Cen Zhang, Sondra Zabar, Steven L. Galetta, Arielle Kurzweil

**Affiliations:** From the Department of Neurology (E.Z., N.A.-F., A.S.L., A.N., S.K.R., C.Z., S.L.G., A.K.), and Department of Medicine (S.Z.), NYU Grossman School of Medicine, New York.

## Abstract

**Introduction and Problem Statement:**

A chief resident's role incorporates administrative, academic, and interpersonal responsibilities essential to managing a successful residency program. However, rising chief residents receive little formal exposure to leadership training.

**Objectives:**

To (1) define leadership styles; (2) understand the effect of cultural competence on leadership styles; (3) learn effective methods to advocate as the chief resident; (4) provide effective peer feedback; (5) provide effective supervisor feedback; (6) learn effective conflict management; (7) ensure psychological safety.

**Methods and Curriculum Description:**

We developed a 1-day curriculum combining didactics and simulation activities for our program's rising chief residents. Implementation of our curricular design included a morning session focusing on small groups and didactic-based lectures on specific topics pertinent to leadership, along with a debriefing of a psychometric evaluation tool administered before the curriculum day. The simulation activity consisted of 3 group objective structured clinical examination (G-OSCE) scenarios: (1) providing a struggling junior trainee with feedback; (2) debriefing an adverse clinical outcome as the team leader; (3) navigating a challenging situation with a supervising physician. Standardized participants were surveyed for specific objectives. Learners completed precurricular and postcurricular surveys on their familiarity and preparedness for their chief year.

**Results and Assessment Data:**

Comparison of preintervention (n = 16) and postintervention (n = 10) data shows improvements in familiarity with leadership models (*p* = 0.006), cultural competence in leadership (*p* = 0.027), and team organizational structure (*p* = 0.010) with notable improvement in report of advocating for the team to 100% of participants in the postcurricular survey. In addition, although not statistically significant, familiarity with specific strategies for feedback delivery improved (*p* = 0.053), as did learner comfort levels with feedback delivery (comparing 51% of learners were either very or somewhat comfortable precurriculum to 90% postcurriculum). This is further supported by standardized participant data after the G-OSCEs. Although familiarity with wellness resources did improve across learners (*p* = 0.421), learner-reported use of wellness resources was noted to be reduced after the curricular intervention and remains a result of further interest for exploration.

**Discussion and Lessons Learned:**

A 1-day leadership development curriculum combining didactics and simulation is an effective means of preparing rising chief residents to succeed in their transition to this leadership role.

## Introduction and Problem Statement

Chief residents play a vital role in managing a successful training program.^1^ Specific administrative, academic, and interpersonal roles are often assigned by program leadership, and a smooth transition to succeed in these individual roles is necessary for reinforcing positive program culture and an overall successful training environment. Despite being tasked with such critical managerial and educational duties, most rising chief residents receive little-to-no formal exposure to management and leadership training before starting their roles.^2,3^ While no published standardized chief resident leadership competencies exist at present, development of a formal curriculum to hone these skills could be of significant benefit to trainees and programs alike.^4-6^

For trainees in neurology programs, the chief residency year serves as the first year of educational and administrative leadership for rising junior faculty members. Although several chief residents go on to assume leadership roles as their careers progress, many become discouraged by the experience of being a chief and may no longer seek opportunities in leadership once training is completed.^7^ Training in interpersonal communication for the situations commonly faced by a rising chief resident is also limited. Interpersonal communication skills and professionalism are difficult to teach, especially when relying solely on lectures and videos.^8^ While various workshops for chief residency training do exist through the American Academy of Neurology and the Accreditation Council for Graduate Medical Education (ACGME), access is often limited to a few participants and the learning opportunities are more passive. Simulations, on the contrary, provide an immersive experience in which the learner interacts with a standardized professional, is directly observed, and is provided with immediate feedback after each station.^9,10^ Simulations are expected to help the learners identify their own learning goals and leave with skills that can be implemented in their new roles.

The overall aim of this study was to develop and evaluate an interactive, experiential-enriched curriculum focused on the development of key leadership, managerial, and interpersonal skills necessary for the successful execution of the chief resident role. The *Know, See, Plan, Do* model helped to structure our combined experiential curriculum, with a focus on acquiring declarative knowledge on leadership. Our learners (the rising chief residents) would be prepared to use their newly acquired skill set (or knowledge) to see or diagnose dynamics in the group or environment and, in so doing, would be prepared to plan a course of action and skillfully intervene to perform the plan (with skills demonstrated using simulation-based and case-based scenarios).^11^ Using the theoretical framework of automaticity and skill expertise, specific leadership concepts and skills are necessary for our rising chief residents to learn before the start of their chief year. Our curriculum focuses on the direct teaching of these skills (associated with the *cognitive phase* of learning, through didactic/small group activities) and observation of our learners practicing these skills in group simulation-based scenarios (*associative phase*), wherein direct feedback is administered by faculty, the standardized participant, and their peers. Further reinforcement of the *autonomous phase* of learning is then observed as the rising chiefs begin their duties and are surveyed about specific frequencies of behaviors.^12^

## Objectives

An experiential enriched curriculum was designed to provide rising chief residents with the skills and knowledge to help them transition smoothly to their new roles. The learning objectives of the curriculum, selected by a focus group of education leaders within our neurology department, were as follows^13-16^:

1. To define key leadership styles (LO#1)
2. To define cultural competence and understand its effect on leadership styles (LO#2)
3. To understand organizational structure and learn effective ways to advocate for your team (LO#3)
4. To provide effective feedback techniques in interaction with peers (LO#4)
5. To provide effective feedback techniques in interaction with supervisors/attending physicians (LO#5)
6. To learn effective ways to manage conflict as a team leader (LO#6)
7. To ensure the psychological safety of team members/participants as a team leader (LO#7)

## Methods and Curriculum Description

### Curriculum Description

We developed a single-day curriculum inclusive of formal didactics followed by case-based simulations. Preceding the start of this intervention, each rising chief resident underwent a psychometric evaluation using the *Insights Discovery* assessment tool, to help understand their leadership style and individual strengths.^17^ We then delivered didactics and workshops to our learners on important topics in leadership. Key topics pertinent to achieve our learning goals/objectives included the following: (1) communication skills, feedback, and crucial conversations (LO# 3, 4, 5); (2) development of a personal leadership model (LO#1); (3) organizational culture and how to advocate for your team within your organization (LO#3); (4) being a culturally competent leader (LO#2), in addition to resources to support personal and team wellness and resilience (LO#7). A debriefing of their psychometric evaluations was conducted in a group format, using communication skills learned in the didactics-based session (LO#1, LO#6).

Didactics were followed by a group objective structured clinical examination (G-OSCE) in our simulation center (NYSIM) that focused on 3 simulated scenarios with attention to maintaining both the learner and the standardized participant's psychological safety: (1) providing a struggling junior trainee with feedback (LO#4, LO#7); (2) debriefing an adverse clinical outcome as the team leader (LO#6, LO#7); (3) navigating a challenging situation with a supervising physician (LO#3, LO#5, LO#7). Each learner had the opportunity to interact with a standardized professional (SP) for 1 case and provide feedback for 2 of the other cases, as a group, to their co-learners, again with attention to the maintenance of psychological safety of all participants (LO#4, LO#5, LO#7). Learners were able to implement the feedback skills learned in the morning didactic session. Each learner received verbal feedback from a faculty observer and written feedback from the standardized participant (SP or “actor”) on his or her performance. The specific case scenarios and their corresponding learning objectives are presented in Table 1.

**Table 1:** Simulation Scenarios and Corresponding Learning Objectives

Topic	Scenario	Learning objectives
1. Providing a struggling junior trainee with feedback	The chief resident learner is running a busy inpatient service and has a PGY2 resident who is not effectively putting in orders, writing timely notes, or picking up new admissions. The PGY2 resident is always on their telephone and seems distracted during the day, especially while on rounds. The chief resident approaches the junior trainee to discuss these concerns. The ultimate concerns discussed pertain to improvement of work-life balance and time management skills	• To provide quality, specific, and direct objective feedback • To provide constructive, actionable feedback • To use the ask-tell-ask model to elicit and provide feedback • To create a nonjudgmental environment • To ensure psychological safety
2. Debriefing an adverse clinical outcome with a treatment team	A 65-year-old man with metastatic melanoma has been hospitalized for 1 wk on the inpatient neurology service. He was on levetiracetam at home for a reported history of seizure. He goes into status epilepticus and is transferred to the medical intensive care unit. On review of records, his medication reconciliation did not correctly identify levetiracetam as a home medication, and it had not been ordered since his admission	• Understand and communicate the facts of the case • Assess team members' reaction to the case outcome • Analyze the process(es) leading to case outcome • Summarize the case, providing take-away points • Create and perform the above process in a nonjudgmental environment • Ensure the psychological safety of team members/participants
3. Navigating a challenging situation with a supervising/attending physician	The chief resident learner is working with a new attending physician who has been managing the service actively, often calling consults, and placing orders on their own. This has made the rotation difficult for the chief resident, who has to advocate for their junior trainees and reinforce incremental autonomy as part of their training environment	• To engage the junior faculty member in a discussion about how the rotation has been going • To professionally and objectively address the junior faculty member's approach • To reinforce resident autonomy as part of our training culture (the junior faculty member is new to this training environment) • To discuss and provide suggestions, along with guidance to the faculty member for future situations • To create a partnership with the junior faculty member in establishing a strong clinical training environment • Ensure the psychological safety of team members/participants

Abbreviation: PGY = postgraduate year.

The session concluded with a team-building/wellness event for all participants (faculty and trainees, LO#7).

### Study Design

One month before the start of the chief year, on the day of our chief resident leadership development day, our rising chief residents were surveyed, using a Likert scale (1 = very unfamiliar, 2 = somewhat unfamiliar, 3 = somewhat familiar, 4 = very familiar), on their (1) familiarity with leadership concepts; (2) readiness/level of preparedness to serve as the chief resident; and (3) levels of comfort in (a) providing feedback to a struggling trainee, (b) debriefing an adverse clinical outcome, and (c) navigating a challenging situation with a supervising physician.

After the completion of the simulation experience, the performance of our rising chief resident was evaluated by the standardized participant (SP or actor) using a behavioral checklist. In addition, a survey of overall level of satisfaction with the simulation experience and the structure of the curriculum was obtained from our rising chief residents, at the completion of the day.

Four months after the completion of the chief resident leadership development day, 3 months into the start of the chief year, our chief residents were again surveyed, using a Likert scale, on their (1) familiarity with leadership concepts; (2) readiness/level of preparedness to serve as the chief resident; and (3) levels of comfort in (a) providing feedback to a struggling trainee, (b) debriefing an adverse clinical outcome, and (c) navigating a challenging situation with a supervising physician.

### Analysis of Data

Descriptive statistics were used to explore the learner's levels of comfort and preparedness across various domains such as delivering feedback and debriefing an adverse clinical event. Given ordinal data from independent groups, precurricular and postcurricular surveys on levels of familiarity with particular leadership concepts were evaluated using the Mann-Whitney *U* test. Quantitative analysis was completed with IBM SPSS version 28.

### Standard Protocols Approvals, Registrations, and Patient Consents

This educational program evaluation met NYU's criteria for certification as a quality improvement project rather than human subject research and was exempt from institutional board review.

### Data Availability

Data not published within this article will be made available on request directed to the corresponding author.

## Results and Assessment Data

### Participants and Descriptive Data

Our study participants included 16 rising chief residents, across our 2 adult neurology residency program tracks (75%), as well as our pediatric neurology (13%) and double-board neuropsychiatry (12%) programs. 56% were male and 44% were female, with a mean age range of 25–34 years. Most residents were in their postgraduate year 3 (75%), with 19% in postgraduate year 4 and 6% in postgraduate year 5. We discuss data on specific curricular objectives in the following, comparing precurricular and postcurricular survey results. Of note, only 10 of our 16 rising chief residents completed the postcurricular survey. Specific demographics are provided in Table 2.

**Table 2:** Demographics of Participants: Comparison Before and After the Curricular Survey

	Precurricular survey participants (N = 16)	Postcurricular survey participants (N = 10)
Age range, y, mean	25–34	25–34
Sex, n (%)		
Female	9 (56)	5 (50)
Male	7 (44)	5 (50)
PGY, n (%)		
PGY3	12 (75)	
PGY4	3 (19)	6 (60)
PGY5	1 (6)	3 (30)
PGY6		1 (10)
Residency program, n (%)		
Adult neurology	12 (75)	6 (60)
Pediatric neurology	2 (12.5)	2 (20)
Double-board (neuro/psych)	2 (12.5)	2 (20)

Abbreviation: PGY = postgraduate year.

Please note that the postgraduate year progressed from precurricular to postcurricular survey collection date because learners advanced to begin their chief year.

Study data were collected and managed using the electronic data capture tool Research Electronic Data Capture, hosted at NYU Grossman School of Medicine.

#### Objective 1: To Define Key Leadership Styles

**Figure 1:**
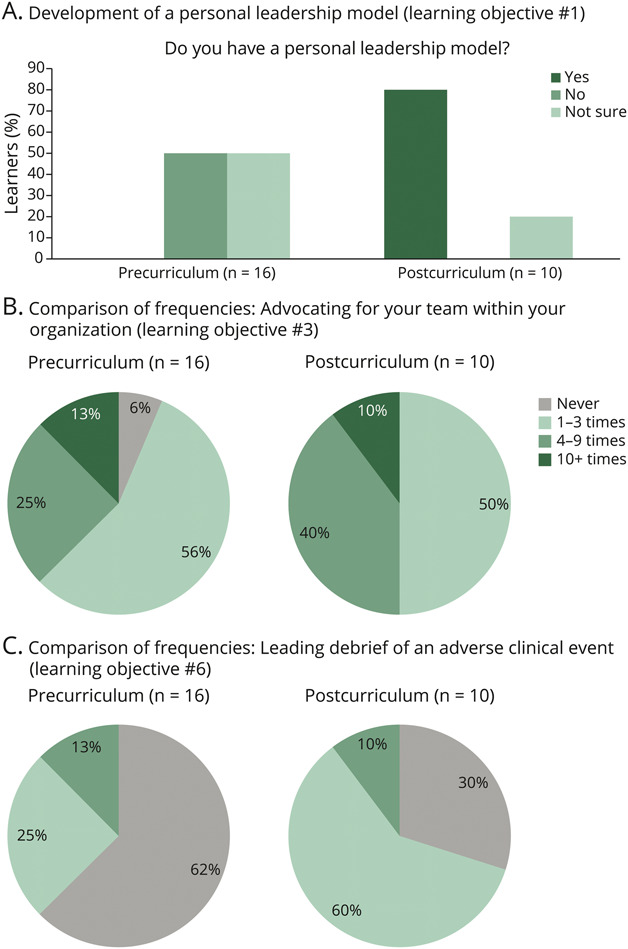
Key Leadership Domains Panel A shows improvement in overall reported frequency of having a leadership model following the curriculum, corresponding to learning objective #1. Panel B shows improvement in the frequency of advocating for one's team within the organization following the curriculum, corresponding to learning objective #3. Panel C shows improvement in the frequency of debriefing an adverse clinical event, corresponding to learning objective #6.

To help our rising chief residents better understand their leadership style and individual strengths, we administered a psychometric evaluation using the *Insights Discovery* assessment tool. In addition, a specific lecture-based didactic was delivered in the morning session of the leadership development day on types of leadership models. Our specific outcome measure was the level of learner familiarity with various leadership models, measured precurriculum and postcurriculum. Precurricular and postcurricular survey results showed that learner familiarity with leadership models improved (comparison of average Likert score 2 [pre] to 3.1 [post], *U*[N_pre_ = 16, N_post_ = 10] = 31.00, *z* = −2.723, *p* = 0.006). In addition, 100% of our learners answered either no (50%) or not sure (50%) when asked whether they had a personal leadership model before the curriculum, with 80% reporting yes to having a leadership model and 20% not sure after the curricular intervention (Figure 1, panel A).

#### Objective 2: To Define Cultural Competence and Understand Its Effect on Leadership Styles

Addressing the importance of cultural competency in leadership, and how this affects leadership styles, was a crucial objective of our curriculum. Our residents received a specific lecture-based didactic in the morning session of the leadership development day on specific aspects of being a culturally competent leader, with a focus on key domains of understanding one's own culture, background, and biases (self-awareness); importance of respect for diversity and knowledge of different cultures; use of effective cross-cultural communication skills (including both verbal and nonverbal communication); discussion of empathy and open-mindedness; and, ultimately, presentation of specific scenarios wherein ones' biases would affect individuals from different cultural backgrounds.^18,19^ The specific outcome measures assessed levels of familiarity with being a culturally competent leader, measured precurriculum and postcurriculum. Precurricular and postcurricular survey results showed that learner familiarity with being a culturally competent leader improved as well (comparison of average Likert score 2.5 [pre] to 3.3 [post], *U*[N_pre_ = 16, N_post_ = 10] = 38.50, *z* = −2.691, *p* = 0.027).

#### Objective 3: To Learn Effective Ways to Advocate for Your Team

Advocating for one's team within an organization is a critical skill for rising leaders to master. To assist our learners in improving their comfort and familiarity levels with being a team advocate, we provided them with a lecture-based didactic on advocating for one's team within an organization delivered during the morning session of the leadership development day, with specific attention to being a team advocate. This included methods in which one's leadership model can function to help achieve this skill. In addition, the learners were able to use their knowledge from the morning session to apply to the simulation encounter with delivering constructive feedback to a faculty member during the G-OSCE. The specific outcome measures assessed levels of familiarity with the team’s organizational structure precurriculum and postcurriculum, as well as our standardized participant data on resident performance during the G-OSCE for “navigating a challenging situation with a faculty member.” Precurricular and postcurricular survey results showed that learner familiarity with the team’s organizational structure improved (comparison of average Likert score 2.4 [pre] to 3.1 [post], *U*[N_pre_ = 16, N_post_ = 10] = 40.50, *z* = −2.560, *p* = 0.010). The majority of our learners (94%) reported a frequency of advocating for their team at least 1–3 times before curricular intervention. One hundred percent of learners reported the behavior of advocating for their organization after curricular intervention (Figure 1, panel B). In addition, SP data from the simulation scenario of navigating a challenging situation with a faculty member revealed that 94% of learners communicated the intention to reinforce resident physician autonomy in clinical decision making (receiving a score of “well done”) and 88% of learners actually went forth with creating a plan and partnering with our SP in reinforcing a strong clinical training environment (receiving a score of “well done”) (Figure 3, panel B).

#### Objective 4: To Provide Effective Feedback Techniques in Interaction With Peers

**Figure 2:**
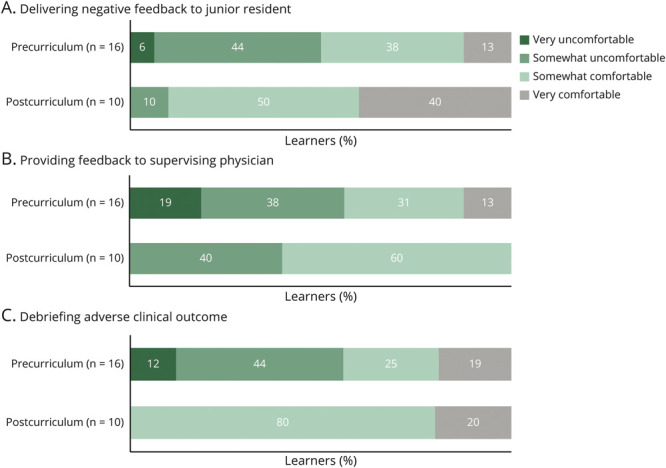
Learner Levels of Comfort Across Simulated Scenarios, Pre- and Postcurriculum Comparison of learner comfort levels precurricular intervention to postcurricular intervention, across our group OSCE scenarios, showing improvement in comfort levels across all three simulated cases.

To assist our rising chief residents in meeting this learning objective, we used both a didactic component in the morning session on effective strategies to give feedback and the opportunity to use these skills in a simulation of a scenario of giving feedback to a struggling junior resident. The specific outcome measures assessed precurricular and postcurricular levels of comfort with delivering feedback to a struggling trainee, familiarity with feedback strategies, and SP data on performance of delivering feedback to a junior resident during the G-OSCE. Evaluation of the changes in familiarity scores with specific methods or strategies of giving feedback showed improvement, although not statistically significant after curricular intervention (comparison of average Likert score 2.8 [pre] to 3.5 [post], *U*[N_pre_ = 16, N_post_ = 10] = 48.00, *z* = −1.938, *p* = 0.053). We also found that comfort in delivering feedback to junior residents improved, with a learner report of very comfortable or somewhat comfortable noted before (51%) and after (90%) curricular intervention (Figure 2, panel A).

**Figure 3:**
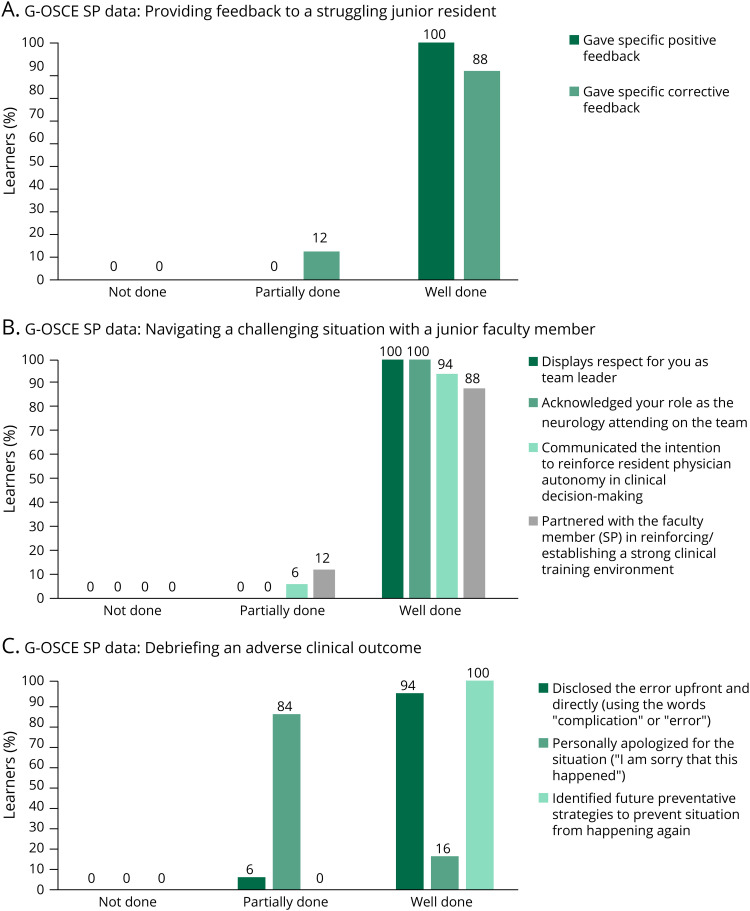
SP Feedback Data for G-OSCE Learner performance on completion of specific tasks pertinent to case objectives by standardized participant categorized into “not done,” “partially done” or “well done” based on predetermined criteria. G-OSCE = group objective structured clinical examination, SP = standardized participant.

SP behavioral survey data for the OSCE of “providing a struggling junior trainee with feedback” revealed that 100% of our learners received full credit (or “well done”) for delivering specific positive feedback and 89% of learners received full credit (or “well done”) for delivering specific corrective feedback (Figure 3, panel A). This demonstrated that the behaviors of effectively using the techniques they learned in the preceding didactic session were implemented in the afternoon simulation session.

#### Objective 5: To Provide Effective Feedback Techniques in Interaction With Supervisors/Attending Physicians

To achieve this learning objective, in addition to our morning didactics on methods and strategies to deliver feedback, we also implemented our G-OSCE on navigating a challenging situation with a faculty member. The specific outcome measures used to assess achievement of this learning objective included learner precurricular and postcurricular levels of comfort in delivering feedback, as well as SP data on performance of delivering constructive feedback to a faculty member during the G-OSCE simulation. The levels of comfort that were reported precurriculum and postcurriculum were notably a more mixed result, with 44% of learners reporting feeling either very comfortable or somewhat comfortable providing feedback to a supervising physician precurriculum and 60% of learners reporting the same postcurriculum. Of interest, 40% of learners remained somewhat uncomfortable with giving feedback to a supervising physician after the curricular intervention (Figure 2, panel B). Some open-ended narrative responses included that the scenario was “less realistic than others” and felt “less commonly encountered than others.”

The SP behavioral checklist data revealed the following in specific behaviors observed: (1) showing respect for them as a team leader (100% full credit, or “well done”); (2) acknowledging their role as a neurology attending on the team (100% full credit, or “well done”); (3) communicating the intention to reinforce resident physician autonomy in clinical decision making (94% full credit, or “well done”); (4) partnering with the faculty member/SP to reinforce a strong clinical training environment (89% full credit, or “well done”) (Figure 3, panel B).

#### Objective 6: To Learn Effective Ways to Manage Conflict as a Team Leader

Implementation of a debriefing on results of the *Insights Discovery* psychometric evaluation tool with our chief resident cohort was used to reinforce this learning objective. Specific attention was given to conflicting personality types within the resident cohort, and techniques to allow for rapid conflict resolution were discussed. We also administered a simulation on debriefing an adverse clinical event as a team leader to allow our rising trainees to practice skills in conflict resolution and mediation. The specific outcome measures assessed precurricular and postcurricular frequencies of debriefing an adverse clinical outcome, self-reported levels of comfort with debriefing an adverse clinical outcome, and, finally, our SP data on the delivery of a medical error during the “debriefing an adverse clinical outcome” G-OSCE. Self-reported frequencies of at least “1–3 times” of debriefing an adverse clinical event improved from 38% precurriculum to 90% postcurriculum (Figure 1, panel C).

Self-reported comfort of our chief resident learners in debriefing an adverse clinical outcome precurriculum and postcurriculum revealed the most significant improvement of the entire curriculum observed in this domain, with 44% of learners reporting feeling either very comfortable or somewhat comfortable precurriculum, compared with 100% of learners reporting the same postcurriculum (Figure 2, panel C).

Once again, our SP behavioral checklist data for the simulated scenario of debriefing an adverse clinical outcome showed that 94% of learners received full credit (or “well done”) in the behavior of disclosing the medical error upfront, directly using the words “error” or “complication,” with 100% of learners identifying future preventative strategies for similar situations. Of interest, only 16% of learners received full credit (or “well done”) for personally apologizing for the situation (Figure 3, panel C).

#### Objective 7: To Ensure the Psychological Safety of Team Members/Participants as a Team Leader

**Figure 4:**
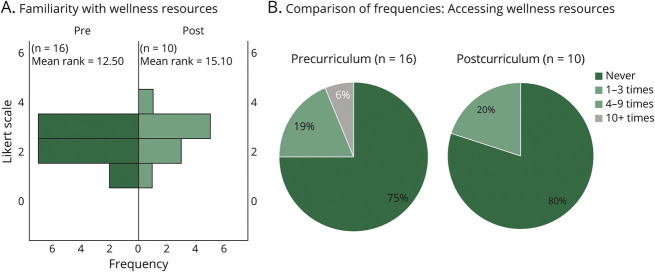
Wellness Objectives Following curricular intervention, there is improvement in familiarity with wellness resources (A), but with reduction in frequency of accessing wellness resources (B).

Our curriculum focused on reinforcing the concept of wellness and psychological safety, of both our learners and their junior trainees. The specific outcome measures assessed precurricular and postcurricular levels of familiarity with institutional wellness resources, as well as comparisons of frequencies of accessing the said wellness resources. Precurricular and postcurricular survey results evaluating the levels of familiarity with institutional, personal, and team wellness resources revealed that only 44% of our learners were somewhat or very familiar with such resources before the leadership development day, with improvement to 100% familiarity after the curricular intervention (70% somewhat familiar, 30% very familiar), although this did not meet statistical significance (*U*[N_pre_ = 16, N_post_ = 10] = 64.00, *z* = −0.918, *p* = 0.421) (Figure 4, panel A).

We also surveyed our chief residents on their self-reported frequencies of accessing these wellness resources. Seventy-five percent of our chiefs reported never having accessed these resources before curricular intervention, but of interest, this frequency increased to 80% after the curricular intervention. This is an important data point, given the cohort of rising chief residents were in their postgraduate year 1 at the peak of the coronavirus disease 2019 pandemic, a time when trainee and faculty wellness resources were widely distributed to members of our institution, and the medical community at large.

Overall, 100% of participants reported satisfaction (either very satisfied or somewhat satisfied) with the pilot of our chief resident leadership development day.

## Discussion and Future Directions

A 1-day interactive chief resident leadership curriculum led to improvements in familiarity across several key leadership domains, including communication skills, feedback, leadership development, organizational culture, and cultural competency. All learners demonstrated improved comfort in simulated case scenarios, with the most prominent improvements noted in debriefing an adverse clinical outcome. Our learners reported satisfaction with all elements of the leadership development day, with the *Insights Discovery* psychometric tool debriefing session noted to be a highlight of the curriculum.

Our G-OSCE format successfully allowed for the completion of the simulation portion of the curriculum within a 1-day time frame. Specific case scenarios that were most successfully simulated included providing feedback to a struggling junior trainee and debriefing an adverse clinical outcome with a treatment team. Our rising chief residents reported that navigating a challenging situation with a supervising physician was the least realistic scenario. Written feedback suggestions included to alter the scenario to include “a hands-off attending physician” or one who is “demonstrating poor professional conduct without awareness of such behavior.”

Our study limitations included a small cohort of learners (n = 16), with fewer participants completing the postcurricular survey (n = 10) in a single academic institution, with absence of a control group in our study design. In addition, recall bias may be implicated in the self-reporting of behavioral frequencies, given our reliance on self-reported data. One piece of intriguing data that merits further exploration is the report of infrequency with which institutional and departmental wellness resources were accessed by our chief resident learners, especially after the curricular pilot. Generalizability of this curriculum across programs should also be considered, with collaborative cross-institutional work encouraged. Assessing the impact of this chief resident leadership curriculum on future program performance across specific content areas of the ACGME survey is another potential future direction. Future iterations of our survey or data collection tools should also undergo validation.^20^

To further assess the impact of our curriculum on the development of specific leadership skills for our chief residents, we are presently collecting ongoing longitudinal survey data of specific self-reported behaviors across leadership domains as reported by our chief residents. We are concomitantly surveying the junior resident (postgraduate years 2 and 3) cohorts along with select supervising faculty across inpatient services, to evaluate how often our chief residents' specific leadership skills are being witnessed in daily clinical, professional, and interpersonal interactions. This data collection is presently underway.

In conclusion, we have demonstrated that a 1-day leadership development curriculum combining didactics and simulation is an effective means of preparing rising chief residents to succeed in their transition to this leadership role. This curriculum is a portable, transferrable method that can be tailored to the specific needs of one's institution and unique chief roles. In so doing, it would allow for a more equitable teaching environment across institutions because the leadership program can implement the specific didactics and include role play for simulation of the various scenarios presented above. Moreover, our leadership curriculum stresses the importance of empowering our senior trainees to begin their chief year as delegates of our training department and further reinforces the significance of their roles as critical in promoting a positive program culture and training environment.

## Appendix Authors

**Table:**

Name	Location	Contribution
Elina Zakin, MD	Department of Neurology, NYU Grossman School of Medicine, New York	Drafting/revision of the manuscript for content, including medical writing for content; major role in the acquisition of data; study concept or design; analysis or interpretation of data
Nada Abou-Fayssal, MD	Department of Neurology, NYU Grossman School of Medicine, New York	Drafting/revision of the manuscript for content, including medical writing for content; study concept or design
Aaron S. Lord, MD, MSc	Department of Neurology, NYU Grossman School of Medicine, New York	Drafting/revision of the manuscript for content, including medical writing for content; study concept or design
Aaron Nelson, MD, MBS	Department of Neurology, NYU Grossman School of Medicine, New York	Major role in the acquisition of data
Sara K. Rostanski, MD	Department of Neurology, NYU Grossman School of Medicine, New York	Major role in the acquisition of data
Cen Zhang, MD	Department of Neurology, NYU Grossman School of Medicine, New York	Major role in the acquisition of data
Sondra Zabar, MD	Department of Medicine, NYU Grossman School of Medicine, New York	Drafting/revision of the manuscript for content, including medical writing for content; study concept or design
Steven L. Galetta, MD	Department of Neurology, NYU Grossman School of Medicine, New York	Drafting/revision of the manuscript for content, including medical writing for content; study concept or design
Arielle Kurzweil, MD	Department of Neurology, NYU Grossman School of Medicine, New York	Drafting/revision of the manuscript for content, including medical writing for content; major role in the acquisition of data; study concept or design

## Glossary

ACGME: Accreditation Council for Graduate Medical Education
G-OSCE: group objective structured clinical examination
SP: standardized professional
